# Assessment of the Risk of Severe Dengue Using Intrahost Viral Population in Dengue Virus Serotype 2 Patients *via* Machine Learning

**DOI:** 10.3389/fcimb.2022.831281

**Published:** 2022-02-10

**Authors:** Su-Jhen Hung, Huey-Pin Tsai, Ya-Fang Wang, Wen-Chien Ko, Jen-Ren Wang, Sheng-Wen Huang

**Affiliations:** ^1^ National Mosquito-Borne Diseases Control Research Center, National Health Research Institutes, Tainan, Taiwan; ^2^ Department of Pathology, National Cheng Kung University Hospital, College of Medicine, National Cheng Kung University, Tainan, Taiwan; ^3^ Department of Medical Laboratory Science and Biotechnology, College of Medicine, National Cheng Kung University, Tainan, Taiwan; ^4^ National Institute of Infectious Diseases and Vaccinology, National Health Research Institutes, Tainan, Taiwan; ^5^ Department of Internal Medicine, National Cheng Kung University Hospital, College of Medicine, National Cheng Kung University, Tainan, Taiwan; ^6^ Department of Medicine, College of Medicine, National Cheng Kung University, Tainan, Taiwan; ^7^ Center of Infectious Disease and Signaling Research, National Cheng Kung University, Tainan, Taiwan

**Keywords:** dengue virus, single nucleotide variants, defective viral genomes, machine learning, severity

## Abstract

Dengue virus, a positive-sense single-stranded RNA virus, continuously threatens human health. Although several criteria for evaluation of severe dengue have been recently established, the ability to prognose the risk of severe outcomes for dengue patients remains limited. Mutant spectra of RNA viruses, including single nucleotide variants (SNVs) and defective virus genomes (DVGs), contribute to viral virulence and growth. Here, we determine the potency of intrahost viral population in dengue patients with primary infection that progresses into severe dengue. A total of 65 dengue virus serotype 2 infected patients in primary infection including 17 severe cases were enrolled. We utilized deep sequencing to directly define the frequency of SNVs and detection times of DVGs in sera of dengue patients and analyzed their associations with severe dengue. Among the detected SNVs and DVGs, the frequencies of 9 SNVs and the detection time of 1 DVG exhibited statistically significant differences between patients with dengue fever and those with severe dengue. By utilizing the detected frequencies/times of the selected SNVs/DVG as features, the machine learning model showed high average with a value of area under the receiver operating characteristic curve (AUROC, 0.966 ± 0.064). The elevation of the frequency of SNVs at E (nucleotide position 995 and 2216), NS2A (nucleotide position 4105), NS3 (nucleotide position 4536, 4606), and NS5 protein (nucleotide position 7643 and 10067) and the detection times of the selected DVG that had a deletion junction in the E protein region (nucleotide positions of the junction: between 969 and 1022) increased the possibility of dengue patients for severe dengue. In summary, we demonstrated the detected frequencies/times of SNVs/DVG in dengue patients associated with severe disease and successfully utilized them to discriminate severe patients using machine learning algorithm. The identified SNVs and DVGs that are associated with severe dengue will expand our understanding of intrahost viral population in dengue pathogenesis.

## Introduction

Dengue virus (DENV), is a mosquito-borne pathogen which causes more than 90 million acute infection cases and 0.5 million fatalities worldwide each year (Bhatt et al., 2013). Dengue virus is transmitted by female mosquitoes mainly of the species *Aedes aegypti* and, to a lesser extent, *Ae. albopictus*. DENV is a member of the genus *Flavivirus* belonging to the Flaviviridae family and has a positive-sense, single-stranded RNA genome with a length of 10.7 kilobases, which encodes a large polyprotein with two untranslated regions at the 5’ and 3’ termini, respectively (Lindenbarch, 2007). Following the polyprotein translation, the translated polyprotein is cleaved into ten mature viral proteins, including three structural proteins: capsid protein (C), pre-membrane/membrane protein (prM/M), and envelope protein (E); and seven non-structural proteins: NS1, NS2A, NS2B, NS3, NS4A, NS4B, and NS5 (Lindenbarch, 2007). The structural proteins play important roles in viral entry into cells, such as E protein in viral attachment, prM/M and E protein in viral fusion, C protein in virion assembly, and prM and E in virus release. According to the antigenic properties mainly contributed by the E protein, DENV has been classified into four serotypes, *i.e.*, DENV-1 to DENV-4, which have recently been found across tropical and subtropical regions worldwide (Roehrig, 2003). The nonstructural proteins have multiple roles in virus replication in hosts, including the assembly of replication complex, immune response modulation, and protease activities (Lindenbarch, 2007).

Dengue is an acute febrile disease; most patients present with acute dengue fever, but 5–20% of patients further progress to severe dengue with bleeding, plasma leakage, shock, organ failure, and death (Gubler, 2002; Khursheed et al., 2013). In the event of an outbreak, patient triage of severe dengue will help clinicians make management decisions concerning those who require hospitalization with intensive care. According to the guidelines set by the World Health Organization (WHO) in 2009 (World Health Organization, 2010), clinical presentations combined with the results of complete blood count tests have been used as markers of dengue severity evaluation; however, the 2009 WHO dengue severity guideline exhibited limited sensitivity in clinical applications (Ajlan et al., 2019), hence may require further improvements. In order to develop more accurate or sensitive method to evaluate dengue disease severity, various studies continuously explored the risk factors associated with DENV infection, including viral and host factors. Dengue viral protein non-structural antigen 1 (NS1) and dengue viremia, combined with immunoglobulin M and immunoglobulin G in plasma, were suggested as viral markers to predict disease outcome (de Mel et al., 2020; Huang S. W. et al., 2020; Martinez-Cuellar et al., 2020). Host factors detected from serum/plasma, saliva, or urine are ideal markers for evaluating disease progression. Numerous categories of biomarkers have been identified for severe dengue prognosis, including cytokines/chemokines, circulating immune response products, endothelial activation molecules, metabolites, circulating cell-free RNAs or microRNAs, and transcriptomic signatures of host genes (Lee et al., 2016; Rathore et al., 2020; Robinson and Einav, 2020).

Intrahost viral population have been sought to potentially influence disease outcome and pathogenesis (Torres et al., 2021). Due to the error-prone nature of RNA polymerase, RNA viruses exhibit high mutation rates, large and diverse population sizes, and fast replication dynamics, which together result in a mutant spectrum with genetically linked variants (Lauring and Andino, 2010). The variants originate from various sources, including single nucleotide variants (SNVs). SNVs functionally cooperate and contribute to the viral fitness and pathogenesis of population in intrahost evolution (Acevedo et al., 2014). In the case of arbovirus infection, studies focusing on chikungunya virus (Coffey et al., 2011; Rozen-Gagnon et al., 2014), West Nile virus (Jerzak et al., 2007; Ciota et al., 2012), and DENV (Wang et al., 2002; Torres et al., 2021) provide information regarding the viral fitness and pathogenesis profiles of individual viral variants. In contrast to the single nucleotide change in SNVs, deletions, insertions, inversions, duplications, and translocations exhibit a larger difference in virus genome. Among them, defective viral genomes (DVGs) may interfere with virus replication by competing for viral or host resources (Li et al., 2011) or by enhancing immune stimulation (Tapia et al., 2013; Sun et al., 2015; Poirier et al., 2018; Linder et al., 2021). Several recent reports demonstrated the appearance of DVGs in natural viruses (Aaskov et al., 2006; Pesko et al., 2012; Sun et al., 2015; Vasilijevic et al., 2017; Li et al., 2018). Most studies demonstrated the negative impact of DVGs on virus replication and production; however, the beneficial effects of DVGs on complete genomes may exist because the optimal proportions of mixtures are more pathogenic than clonal wild type populations (Simon et al., 2006). Intrahost viral evolution study of DENV has indicated that the viral mutant spectrum was shaped by various selection pressures in human DENV infection, such as immune selection pressures, tissue tropism, or replication defects (Parameswaran et al., 2017). As DENV was transmitted to human host by *Aedes* vectors, we hypothesized that the mutant spectrum of DENV, including SNVs and DVGs, might provide valuable viral genetic information that associated with viral fitness/pathogenesis within the viral transmission cycles between human hosts and *Aedes* species, which implied its possible correlation to disease severity.

Recently, machine learning (ML) algorithms have been used to improve the results as classifying or predicting the disease outcomes. ML, a scientific discipline that focuses on how computers learn from data, has recently been applied in various fields of health and medicine. It combines the intersection of statistics and efficient computing algorithms to analyze massive data sets for various purposes (Deo, 2015). Recent studies have utilized ML for various applications, including image recognition, patient phenotyping, and outcome prediction for a variety of human diseases (Nanayakkara et al., 2018; Lee and Lee, 2020; Abadir et al., 2020; Huang Y. et al., 2020; Hugle et al., 2020; Islam et al., 2020; Jamal et al., 2020; Mekov et al., 2020; Milanez-Almeida et al., 2020). In combination with genetic, metabolomic, and physical data from patients, ML has been utilized for diagnosis, prognosis, and prediction of hospitalization risk in arbovirus infectious diseases, such as dengue and zika viruses (Melo et al., 2018; Davi et al., 2019; Nagori et al., 2019; Ho et al., 2020; Sippy et al., 2020). The advantages of ML include improved medical treatment of patients and reduced duration of diagnosis using medical imaging or laboratory tests.

In our previous study, we developed a rapid triage model for severe dengue using an ML algorithm based on the demographic information and dengue antigen/antibody rapid test results (Huang S. W. et al., 2020). Our developed model showed average good discrimination performance; however, prediction results of the patients, who were suspected to be primarily infected without anti-dengue antibody detectable by antibody rapid tests, are most likely to be false-negative. Therefore, to define other risk factors correlated with disease severity of primary infection patients, we determined the association of SNVs/DVGs with severe dengue in dengue patients without pre-existing antibodies upon their arrival at hospital. By using an unbiased deep sequencing method to determine the intrahost viral population, we retrospectively identified the DENV SNVs and DVGs in the sera of the patients, and this was statistically associated with disease outcomes. We applied the ML algorithm and developed an accurate model to discriminate severe patients using the identified SNVs and DVGs. Also, we performed model explanation to define the importance of SNVs and DVGs in the developed model. Through this study, we demonstrated the diverse profile of SNVs and DVGs in severe dengue patients, which may provide biological information that we can learn about DENV infection and severity.

## Materials and Methods

### Ethics Statement

Our study has been approved by Institutional Review Board of National Cheng Kung University Hospital (approval no. A-ER-106-133 and B-ER-107-244) as the informed consent cannot be achieved due to the anonymized clinical samples. All sera and clinical data were anonymized and de-identified.

### Clinical Characteristics of Patients With Dengue

Suspected DENV-infected patients were enrolled at the National Cheng Kung University Hospital between July and November 2015, as described earlier (Tsai et al., 2016). To focus on the suspected primary infection patients, we selected 65 patients who were dual negative for anti-dengue IgM and anti-dengue IgG. Molecular tests for DENV serotyping were performed, and all patients involved in this study were found to be infected with DENV-2. The 65 selected patients were categorized as having mild (48/65) or severe (17/65) dengue according to the 2009 WHO criteria for dengue severity. Dengue cases that fulfilled one of the following 2009 WHO criteria were categorized as severe: severe plasma leakage, severe bleeding, or severe organ involvement; other cases were categorized as mild. For DENV viral loads, viral RNA copies were determined using LightMix dengue virus EC (TIB MOLBIOL GmbH, Germany) quantitative reverse transcription polymerase chain reaction (qRT-PCR), as described earlier (Tsai et al., 2016). NS1 antigen and anti-dengue IgM and IgG antibodies were examined using one-step immunochromatographic assay by Dengue Duo Dengue NS1 Ag + Ab Combo assays (SD BIOLINE, Yongin, Korea) for antigen and antibody detection.

### Unbiased Deep Sequencing for SNVs and DVGs Detection of DENV 2

Although DENV genome sequencing by utilizing multiple overlapping amplicons was widely applied, designing primers in specific gene loci can introduce sequencing bias information, especially in the case of DVGs. Therefore, to avoid bias towards known strains and variants without capturing the divergent or unknown variants, we applied the unbiased deep sequencing method, previously described by Matranga, et al., to generate intrahost variant calls from patients’ sera (Matranga et al., 2016). Due to the limited amount of specimens available, we extracted dengue viral RNA from 10 μL of patient sera using a QIAmp Viral RNA Mini Kit (QIAGEN, Germany) according to manufacturer’s instructions. In brief, the extracted RNA (55 μL) was treated with Turbo DNase (Invitrogen, United States) and purified using RNAClean XP beads (Beckman Coulter, United States) to remove contaminating cellular DNA. We depleted contaminating poly(rA) carrier (which is widely utilized to enrich extracted viral RNA during RNA extraction) and ribosomal RNA from human samples using oligo (d)T and 95 ribosomal RNA specific probes with RNase H treatment and purified them using RNAClean XP (Beckman Coulter, United States). A depletion process was considered to improve the quality of viral RNA reads to prepare unbiased total RNA sequencing libraries. To increase the amount of viral cDNA obtained from limited viral RNA to achieve the recommended amount of cDNA for deep sequencing library preparation, we further utilized the Ribo-SPIA^®^ method to synthesize cDNA from RNA (Ovation RNA-Seq System V2, NuGEN, USA) according to the manufacturer’s instructions. In the Ribo-SPIA^®^ method, a DNA/RNA chimeric SPIA primer, DNA polymerase, and RNase H were used in a homogeneous isothermal assay that provides highly efficient amplification of cDNA. The yield of amplified cDNA was quantified using a Qubit Fluorometer (Invitrogen, United States). The deep sequencing libraries were employed using the TruSeq^®^ Nano DNA Library Prep (Illumina, United States), and 9 to 18 cycles of PCR amplification of the libraries were performed according to the manufacturer’s instructions. Finally, libraries were pooled in equal molar amounts and were paired-end sequenced in a HiSeq 4000 or a Nextseq 500 at Genomics Inc. (Taiwan). We used bcl2fastq2 v2.20 (Illumina, United States) to demultiplex sequencing reads and Trimmomatic v0.36 (Bolger et al., 2014) to trim adaptor sequences from sequencing reads. For each serum, we subsequently filtered the DENV-2 reads using the D2/Taiwan/704TN1505a/2015 strain (GenBank accession number: KU365901), which was isolated in the same outbreak and sequenced by Sanger dideoxy sequencing. Afterwards, the DENV-2 reads were *de novo* assembled using Trinity (Grabherr et al., 2011), and the contigs were scaffolded using the VectorNTI program v9.0 (Invitrogen, United States). The *de novo* assembled consensus sequence was used as a reference sequence. Burrows–Wheeler Aligner (Li and Durbin, 2009) and TopHat2 v2.1.1 (Kim et al., 2013) were used to align the reads to consensus sequence and to define DVGs existing in the viral population, respectively. In SNVs detection, we utilized LoFreq (Wilm et al., 2012) to detect the occurrence of SNVs among the mapped reads. Instead of using a minimum threshold to distinguish the sequencing noise and true variants in SNV calling, LoFreq library identified variant positions marked by a significant bias in the strand from which the supporting reads are derived. LoFreq library does so by doing a two-tailed Fisher’s exact test of the hypothesis that variant-base forward and reverse strand counts come from the same distribution as the consensus base. SNVs were called with a Bonferroni-corrected P-value threshold of 0.05, and SNVs with high strand bias (low P-value; Holm–Bonferroni corrected for multiple-hypothesis testing) were ignored from LoFreq predictions. The frequencies of SNVs and the detection times of DVGs were considered zero when the abundance of SNVs and DVGs was undetectable in our variant analysis pipelines. The deep sequencing reads of DENV-2 were deposited in the NCBI Sequence Read Archive (accession number SRR16924914 and SRR16943989 to SRR16944052) under the BioProject ID PRJNA779757.

### Phylogenetic and Statistical Methods

By using the *de novo* assembled consensus sequences retrieved from patient sera by deep sequencing, we aligned 65 consensus sequences with the indicated DENV-2 sequences published in the GenBank database and constructed the Maximum likelihood phylogenetic trees with 1000 bootstrap replicates using MEGA v11.0 (Tamura et al., 2021). To evaluate the association between the SNVs/DVGs identified in the sera of patients with suspected dengue and the prognosis of severe dengue, we first employed Shapiro–Wilk test in SciPy library v1.7.1 to examine the data normality of each SNV and DVG. Since most of the SNVs and DVGs except 3 SNVs were not drawn from a normal distribution, we applied the Mann–Whitney U test in SciPy library to examine whether the distributions of mild and severe dengue groups are equal. Additionally, Spearman’s rank correlation method in pandas library v1.3.4 was employed to examine the correlation coefficient between the indicated SNVs/DVGs and disease severity. Chi-square test was utilized to analyze the association between identified SNV clusters and the genes where SNVs located.

### ML Model Development and Explanation

We performed extreme gradient boosting machine (XGBoost) using XGBoost 1.5.0 (Chen and Guestrin, 2016), which is an optimized distributed gradient boosting library designed to be highly efficient, flexible, and portable, to develop the prediction ML model. XGBoost is an optimized distributed gradient boosting algorithm that provides parallel tree boosting to rapidly and accurately solve problems associated with scientific data (Chen and Guestrin, 2016). Models were then constructed using open-source software libraries, including Scikit-learn 0.22.2 (Pedregosa et al., 2011) and Python 3.7 (Pilgrim, 2009).

We used a stratified 10-fold cross-validation approach with training and testing datasets to validate the performance of the models. In our approach, we randomly partitioned the mild and severe cases into 10 subsets, each of which had equal numbers of the mild and severe cases. Of the 10 subsets, a single subset was retained as the validation data for testing the model, and the remaining 9 subsets were used as training data for ML model development. We then repeated this process 10 times (the folds), with each of the 10 subsets being used as the validation data, exactly once. In each instance of partition, the remaining training data was used to develop the ML model.

To fine-tune the parameters, we conducted hyperparameter searches using Optuna library (Akiba et al., 2019) to search for optimal hyperparameters for the ML model. Due to the limited number of patients who were enrolled and the data imbalance between mild and severe dengue groups in this study, the area under the Precision-Recall curve was referred to as the optimization metric. Ten-fold cross-validation across the training set was also used, and the data were randomly partitioned 9:1 into training and testing sets with ten replicates. The area under the Precision-Recall curve of the ML model was assessed using the logarithmic loss function. Full hyperparameter search ranges and final model hyperparameters are available in the online repository (https://github.com/joehuang1980/SNV_DVG).

To identify potentially relevant features on a per-patient basis, we assessed the explainability using SHapley Additive exPlanations (SHAP). Briefly, the SHAP method, which has been previously described in detail (Lundberg and Lee, 2017), connects game theory with local explanations, uniting several previous methods. SHAP generates a locally interpretable model for individual predictions from a complex model using an explainer method that combines the inputs together to evaluate the effects on the predictive model.

## Results

### DENV Mutant Spectra in SNVs and DVGs From Patient Sera Using Unbiased Deep Sequencing

**Figure 1:**
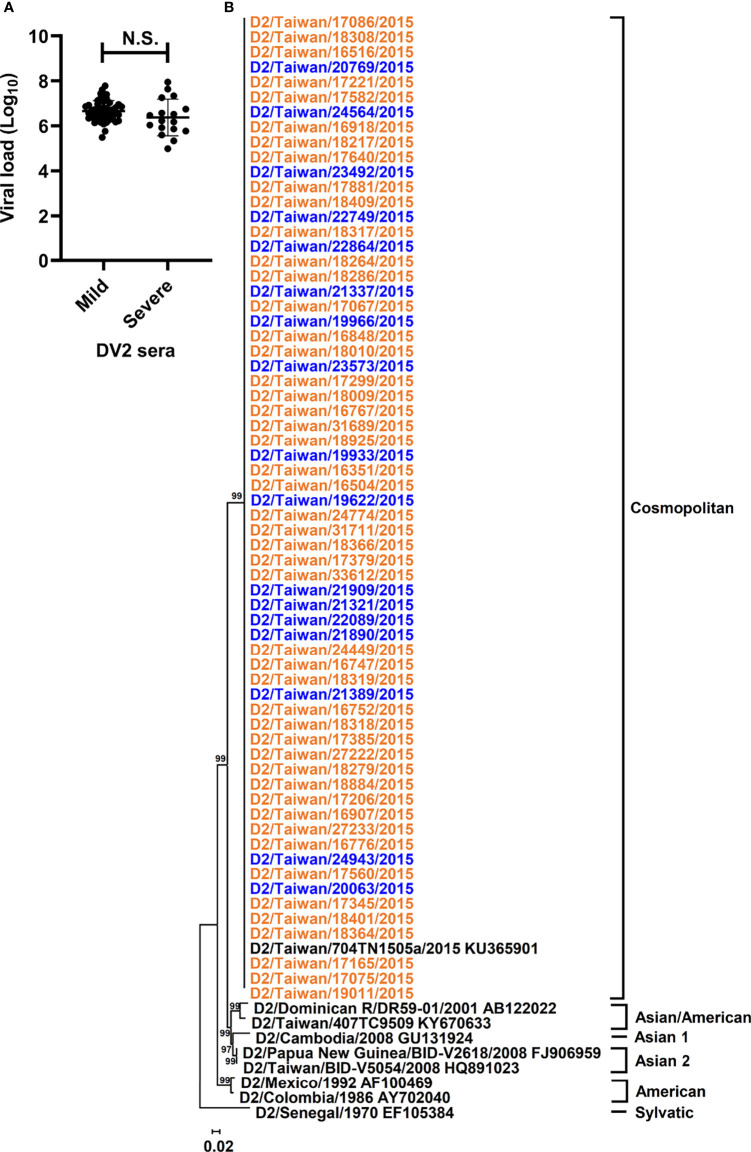
Viral loads and phylogeny of DENV-2 sequences from patients with different disease severity. Viral loads and phylogeny of viral consensus sequences from DENV-2 patients with diverse disease outcomes were displayed. **(A)** The viral loads in the sera of DENV-2 infected patients with mild and severe disease were compared. N.S. indicated p > 0.05 in Mann-Whitney U test. **(B)** Consensus sequences of polyprotein coding region from the sera of enrolled DENV-2 patients and those of DENV-2 prototypes were analyzed using the maximum likelihood method with 1000 bootstrap replicates. Sequences from mild cases (orange) and severe cases (blue) were indicated by different colors and clustered into 6 genotypes, including Sylvatic, American, Asian 1, Asian 2, Asian/American, and Cosmopolitan genotypes. The bootstrap values of each genotype were indicated at the root of the branches.

All sera in this study were collected on the first day of arrival at the hospital from 65 individuals, who were primarily infected by DENV-2 according to the undetectable anti-DENV antibody by rapid test, during the 2015 dengue outbreak in Tainan city, Taiwan (Tsai et al., 2016). To determine whether the quantitative or qualitative patterns of dengue genomes in sera are associated with the disease outcomes, we first examined the quantity of the RNA copy numbers in the sera of patients belonging to mild and severe dengue groups using qRT-PCR. Regarding the abundance of viral RNA copies, viral RNA amount in the collected sera ranged from 9.7 × 10^4^ to 8.8 × 10^7^ copies (**Supplementary Table 1**), and no significant difference was found between mild and severe dengue groups (**Figure 1A**) by using the Mann–Whitney U test, which suggested that DENV genomes were present in similar amounts in the sera of mild and severe dengue patients.

To analyze different qualitative patterns of DENV genomes, we used unbiased deep sequencing to simultaneously determine the whole genome consensus sequences as well as intrahost SNVs and DVGs of the DENV population. The total numbers of obtained reads which were aligned to DENV-2 reference strain ranged from 1.8 × 10^5^ to 1.8 × 10^7^ with 6.2 ± 4.1 × 10^6^ reads (**Supplementary Table 1**), and the whole genome consensus sequences, which represented the interhost population genomes from each patient, were *de novo* assembled using the reads. To determine whether the consensus sequences from severe dengue patients showed diverse genetic profiles in contrast with those from mild patients, we performed phylogenetic analysis to determine the phylogeny of the dengue sequences from collected sera. Along with the sequences of DENV-2 prototype strains, phylogenetic results showed that all sequences were clustered together and belonged to the genotype Cosmopolitan of DENV-2 (**Figure 1B** and **Supplementary Figure 1**). In addition, consensus sequence alignment result identified 177 nucleotide mutations; however, no mutation was defined to associate with the disease severity (**Supplementary Table 2**). Therefore, the interhost populations of DENV genomes among all the dengue patients were suggested to be closely related without diversely clustering along with different disease outcomes.

### Defining DENV Mutant Spectra in SNVs and DVGs Associated With Dengue Severity

**Figure 2:**
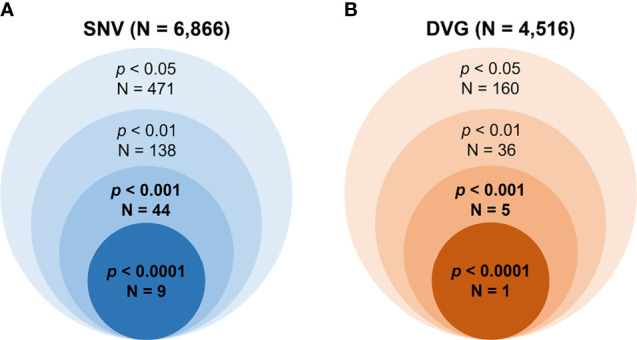
Summary of the numbers of identified SNVs/DVGs that associated with dengue disease outcomes. The identified numbers of SNVs **(A)** and DVGs **(B)** by using different thresholds of p value with Mann–Whitney U test were summarized. A total of 6,866 SNVs and 4,516 DVGs were identified from 65 enrolled DENV-2-infected patients, respectively. In the Mann–Whitney U test results, forty-four SNVs and five DVGs were observed to be highly associated with disease severity in DENV-2-infected patients, with p < 0.001, and nine SNVs and one DVG, with p < 0.0001.

Mutant spectra of RNA viruses have been reported to be associated with viral fitness and pathogenesis (Wang et al., 2002; Ciota et al., 2012; Rozen-Gagnon et al., 2014). Since the interhost DENV-2 consensus genomes did not show the association of disease outcomes, we assessed the intrahost population of DENVs, including SNVs and DVGs obtained from sera of DENV-2 patients, to define their associations with severe dengue group. First, we defined the SNV frequencies and detection times of DVGs which appeared in intrahost dengue population using LoFreq (Wilm et al., 2012) and TopHat2 (Kim et al., 2013) programs, respectively. A total of 6,866 SNVs and 4,516 DVGs were defined from the sera of 65 patients (**Figure 2**). Next, we applied statistical methods to identify the SNVs and DVGs showing high potential to be associated with disease severity. We denominated the SNVs according to their nucleotide position combining with sequence change and the DVGs according to the nucleotide start and end position of the deleted junction region, respectively. To choose statistical methods for correlation analysis, we first assessed the distribution of SNVs and DVGs between mild and severe patients by the Shapiro–Wilk test. Most SNV frequencies and detection times of DVGs did not follow a normal distribution except for 3 SNVs (**Supplementary Table 3**). Thus, we next used the Mann–Whitney *U* test, a non-parametric hypothesis test, to determine the SNVs and/or DVGs associated with disease severity.

**Figure 3:**
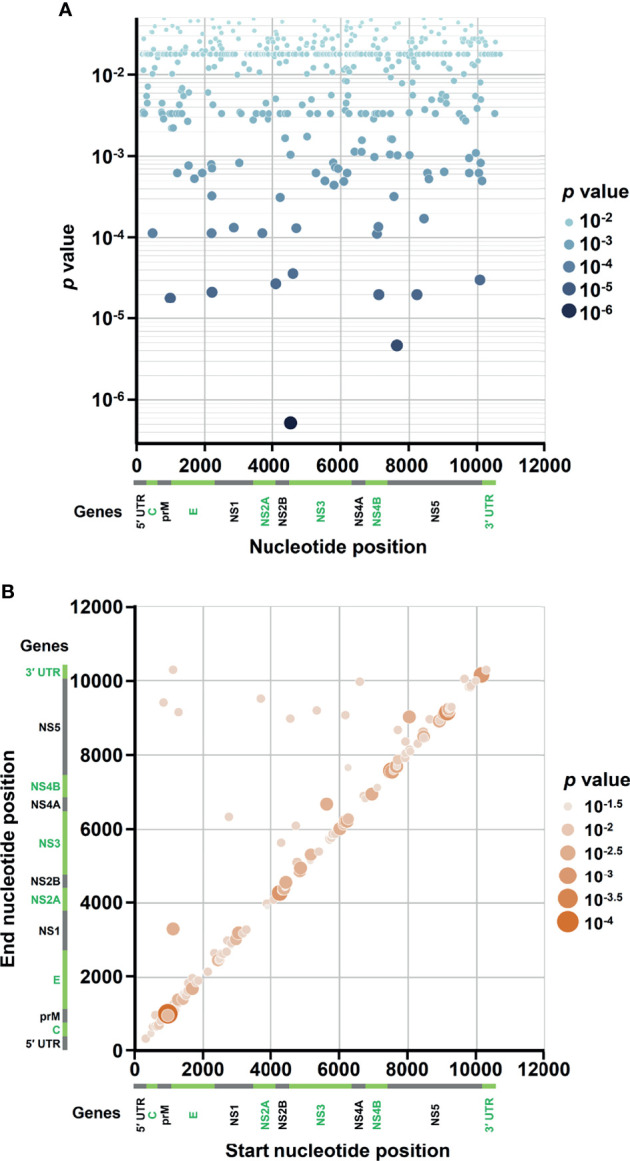
Occurrence map of SNVs/DVGs associated with dengue disease outcomes in DENV-2 genome. Based on the nucleotide positions in DENV-2 genome, the occurrences of **(A)** 471 SNVs and **(B)** 160 DVGs in enrolled DENV-2-infected patients with *p* < 0.05 obtained using Mann–Whitney U test are shown. **(A)** SNVs dot plot mapping nucleotide positions (x-axis) and the *p* values of the association between each SNV and disease severity (y-axis), which assigns *p* values to continuous color and discrete size. **(B)** DVGs dot plot mapping the start and end positions of each junction region of the DVGs, which assigns *p* values of the association between each DVG and disease severity to continuous color and discrete size.

**Figure 4:**
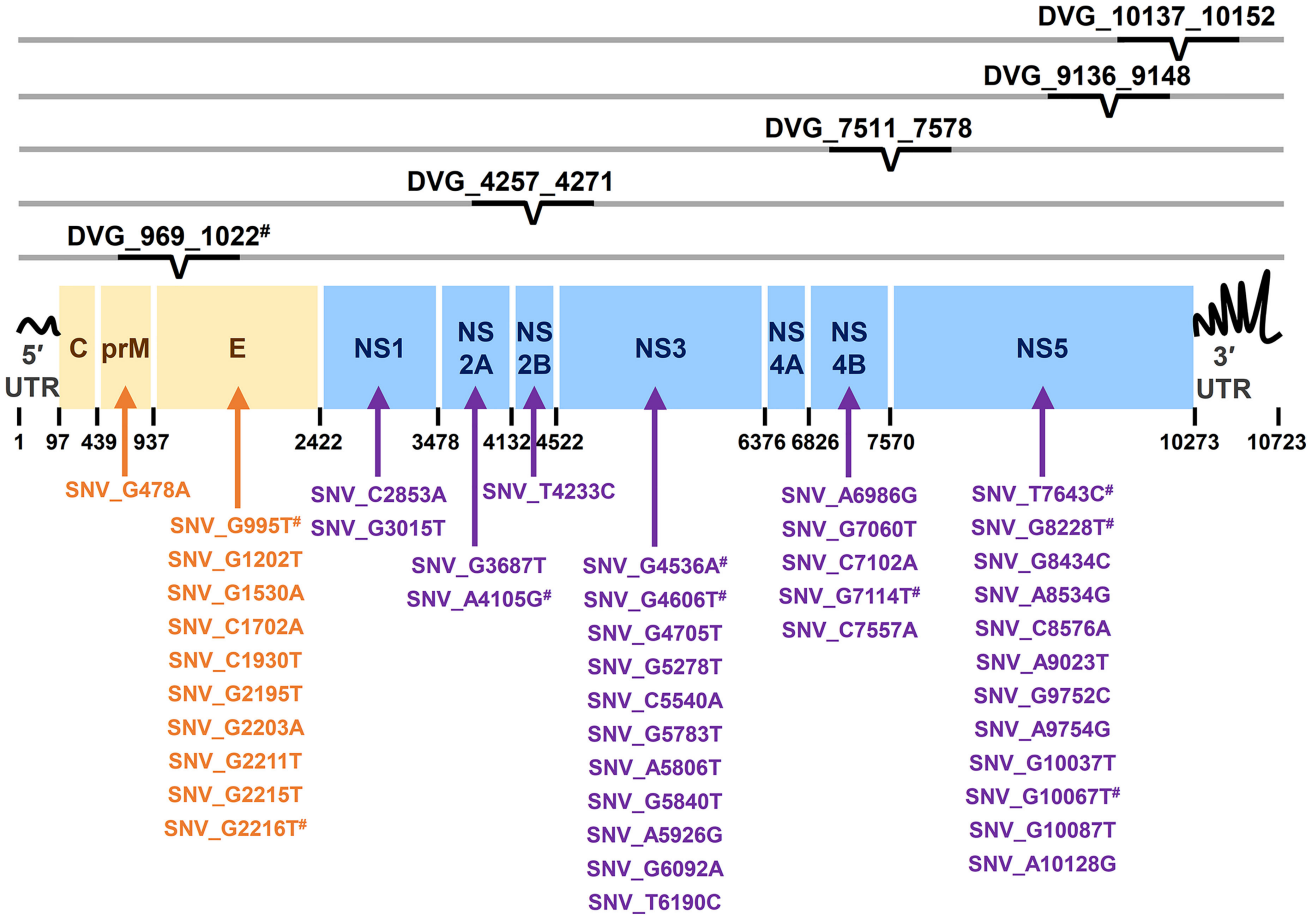
Summary of location distribution of SNVs/DVGs which exhibited high association with dengue disease outcomes in DENV-2 genome. The locations of the nucleotide positions of 44 SNVs and the junction regions of five DVGs that were highly associated with disease severity in DENV-2-infected patients with *p* < 0.001 are shown in DENV-2 genome. The structural protein (yellow blocks) and non-structural protein (blue blocks) coding regions as well as viral protein coding genes are shown. Arrows indicate the protein coding genes in which the SNVs are located. # indicated the SNVs/DVG which exhibited *p* < 0.001 in Mann-Whitney U Test.

In the SNVs profile, a total of 471 SNVs showed significantly different frequency distributions in the two disease severity groups (Mann–Whitney U test, *p* < 0.05, two-tailed) (**Figure 2**). Instead of appearing in certain genes, the identified SNVs located through the DENV-2 genome (**Figure 3A**). Among the identified SNVs, a total of 138, 44, and 9 SNVs exhibited *p*-values of *p* < 0.01, *p* < 0.001, and *p* < 0.0001 in Mann–Whitney U test, respectively (**Figure 2**). The statistical difference did not result from the diverse number of viral copies or the sequencing reads in sera obtained from patients with mild and severe outcomes used as an input for generating sequencing libraries (**Supplementary Table 1**) because these factors were comparable between mild and severe dengue patient groups. Among the 44 SNVs with *p* < 0.001, 11 (25%) and 33 (75%) SNVs were respectively located in structural proteins and non-structural proteins, including 1 SNV located in prM, 10 SNVs in E, 2 SNVs in NS1, 2 SNVs in NS2A, 1 SNV in NS2B, 11 SNV in NS3, 5 SNVs in NS4B, and 12 SNVs in NS5 (**Figure 4**). We did not identify any SNV, which highly associated with disease outcomes of DENV-2 patients, located in untranslated regions (UTRs). We further examined whether the SNVs were non-synonymous mutations that changed the amino acid sequence. Results showed that 88.6% (39 in 44 SNVs) of SNVs with *p* < 0.001 and 100% of SNVs with *p* < 0.0001 resulted in amino acid substitutions in viral proteins (**Table 1** and **Supplementary Figure 2**). In addition, 93.1% (41 in 44 SNVs) displayed higher detected frequency in severe patients than mild ones. Only SNV_G1530A, SNV_T4233C, and SNV_G9752C exhibited higher detected frequency in mild patients (**Supplementary Figure 2**).

**Table 1:** SNVs associated with disease severity in patients with DENV-2 infection.

Gene	SNV^a^	Nucleotide position^b^	Amino acid position^b^	Nucleotide change	Amino acid change	*p* value^c^
**pr**						
	SNV_G478A	478	128	G → A	V → I	1.13×10^-4^
**E**						
	SNV_G995T^#^	995	300	G → T	W → L	1.8×10^-5^
	SNV_G1202T	1202	369	G → T	R → I	6.21×10^-4^
	SNV_G1530A	1530	478	G → A	L → L	7.67×10^-4^
	SNV_C1702A	1702	536	C → A	Q → K	5.29×10^-4^
	SNV_C1930T	1930	612	C → T	P → S	6.21×10^-4^
	SNV_G2195T	2195	700	G → T	W → L	7.92×10^-4^
	SNV_G2203A	2203	703	G → A	G → R	1.13×10^-4^
	SNV_G2211T	2211	705	G → T	L → F	3.25×10^-4^
	SNV_G2215T	2215	707	G → T	G → W	7.1×10^-4^
	SNV_G2216T^#^	2216	707	G → T	G → V	2.13×10^-5^
**NS1**						
	SNV_C2853A	2853	919	C → A	P → P	1.33×10^-4^
	SNV_G3015T	3015	973	G → T	M → I	8.26×10^-4^
**NS2A**						
	SNV_G3687T	3687	1197	G → T	M → I	1.13×10^-4^
	SNV_A4105G^#^	4105	1337	A → G	T → A	2.72×10^-5^
**NS2B**						
	SNV_T4233C	4233	1379	T → C	A → A	3.11×10^-4^
**NS3**						
	SNV_G4536A^#^	4536	1480	G → A	W → stop	5.22×10^-7^
	SNV_G4606T^#^	4606	1504	G → T	G → W	3.62×10^-5^
	SNV_G4705T	4705	1537	G → T	G → W	1.31×10^-4^
	SNV_G5278T	5278	1728	G → T	G → W	6.21×10^-4^
	SNV_C5540A	5540	1815	C → A	P → H	4.96×10^-4^
	SNV_G5783T	5783	1896	G → T	R → M	8.3×10^-4^
	SNV_A5806T	5806	1904	A → T	M → L	4.4×10^-4^
	SNV_G5840T	5840	1915	G → T	R → L	7.29×10^-4^
	SNV_A5926G	5926	1944	A → G	N → D	7.01×10^-4^
	SNV_G6092A	6092	1999	G → A	R → H	4.89×10^-4^
	SNV_T6190C	6190	2032	T → C	Y → H	6.21×10^-4^
**NS4B**						
	SNV_A6986G	6986	2297	A → G	H → R	9.75×10^-4^
	SNV_G7060T	7060	2322	G → T	G → W	1.11×10^-4^
	SNV_C7102A	7102	2336	C → A	L → I	1.35×10^-4^
	SNV_G7114T^#^	7114	2340	G → T	G → W	1.99×10^-5^
	SNV_C7557A	7557	2487	C → A	A → A	3.19×10^-4^
**NS5**						
	SNV_T7643C^#^	7643	2516	T → C	F → S	4.64×10^-6^
	SNV_G8228T^#^	8228	2711	G → T	W → L	1.99×10^-5^
	SNV_G8434C	8434	2780	G → C	E → Q	1.71×10^-4^
	SNV_A8534G	8534	2813	A → G	N → S	6.21×10^-4^
	SNV_C8576A	8576	2827	C → A	P → H	5.24×10^-4^
	SNV_A9023T	9023	2976	A → T	E → V	6.42×10^-4^
	SNV_G9752C	9752	3219	G → C	C → S	9.47×10^-4^
	SNV_A9754G	9754	3220	A → G	R → G	6.21×10^-4^
	SNV_G10037T	10037	3314	G → T	W → L	6.21×10^-4^
	SNV_G10067T^#^	10067	3324	G → T	W → L	3.03×10^-5^
	SNV_G10087T	10087	3331	G → T	G → W	8.26×10^-4^
	SNV_A10128G	10128	3344	A → G	L → L	4.96×10^-4^

a The name of SNVs were assigned according to their nucleotide position and sequence change.

b The nucleotide and amino acid numbering are referred to D2/Taiwan/704TN1505a/2015 strain (GenBank accession number: KU365901).

c by Mann–Whitney U test.

^#^p value < 0.0001.

In the DVGs profile, we defined a total of 4,516 DVGs containing diverse deletion junctions. Among all the identified DVGs, we found that 160 DVGs exhibited significantly different patterns in the two disease severity groups (Mann–Whitney, *p* < 0.05, two-tailed) (**Figure 2**). Among the identified DVGs, a total of 36, 5, and 1 DVGs exhibited *p*-values of *p* < 0.01, *p* < 0.001, and *p* < 0.0001 in Mann–Whitney U test, respectively (**Figure 2**). To determine whether the deletion junctions of DVGs were enriched in specific regions in the DENV genome, we mapped the DVGs according to their start and end nucleotide position. Similar to the location distribution of SNVs, we did not observe the deletion junction of DVGs accumulating in specific genome regions (**Figure 3B**). Moreover, we did not define the DVGs that highly associated with disease outcomes of DENV-2 patients containing deletion in UTRs. Among the identified DVGs, 5 DVGs exhibited *p* < 0.001 and only DVG_969_1022 (with deletion in E gene from nucleotide position 969 to 1022) showed *p* < 0.0001 in Mann–Whitney *U* test, respectively (**Figure 4**), suggesting their high association with disease severity in patients with DENV infection. In addition, we found higher detected times of 5 DVGs in severe than mild patients (**Supplementary Figure 2**). When we further analyzed whether the DVGs change the reading frame, all the five DVGs resulted in reading frame shifts or translation stop down-stream of the deletion junction (**Table 2**), suggesting that the DVGs contained substantial defects in translating the viral proteins, especially those located after the deletion junction of DVGs.

**Table 2:** DVGs associated with disease severity in patients with DENV-2 infection.

Gene region of junction	DVG^a^	Start nucleotide position	End nucleotide position	Junction length	*p* value^b^
**E**					
	DVG_969_1022	969	1022	54	8.86×10^-5^
**NS2B**					
	DVG_4257_4271	4257	4271	15	8.56×10^-4^
**NS4B_NS5**					
	DVG_7511_7578	7511	7578	68	5.84×10^-4^
**NS5**					
	DVG_9136_9148	9136	9148	13	6.21×10^-4^
	DVG_10137_10152	10137	10152	16	9.82×10^-4^

a DVGs were assigned according to their nucleotide start position and end position of deleted junction regions.

b Using Mann–Whitney U test.

**Figure 5:**
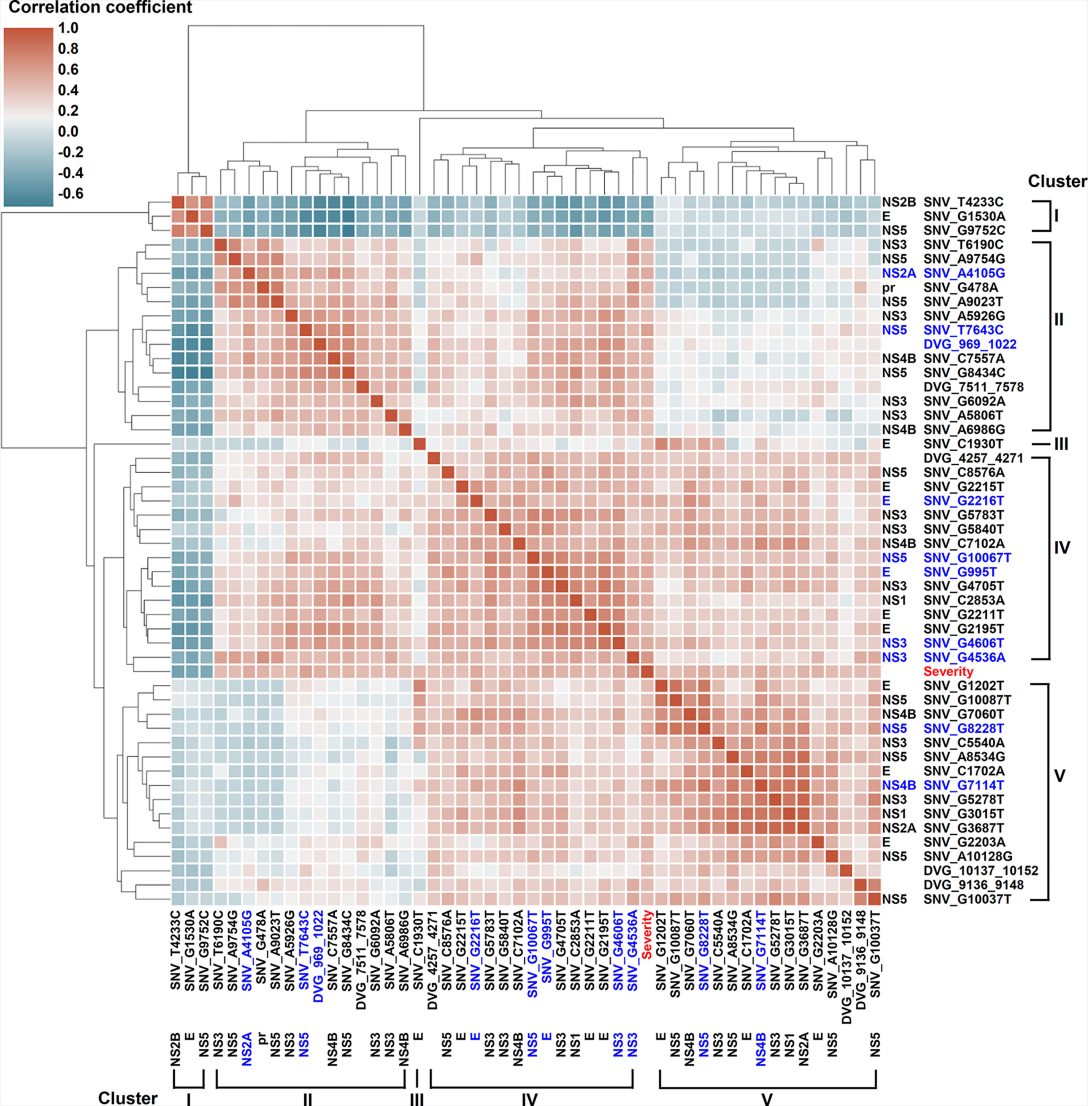
Interactive correlation of SNVs/DVGs highly associated with disease severity in DENV-2-infected patients. The 44 SNVs and the junction regions of 5 DVGs highly associated with disease severity in DENV-2-infected patients with *p* < 0.001 in Mann–Whitney U test were selected in the plot. The 9 SNVs and the junction regions of 1 DVG with *p* < 0.0001 in Mann–Whitney U test were highlighted in blue. The correlation coefficient of each SNVs/DVGs pair was evaluated by the Spearman’s rank correlation method and displayed using hierarchically-clustered heatmap. Heatmap assigns a continuous color for correlation coefficients, and hierarchical cluster groups the SNVs/DVGs exhibiting similar profiles into clusters.

We next assessed the interactive correlation between each selected SNV/DVG and other selected SNVs/DVGs using a hierarchically-clustered heatmap (**Figure 5**). In this heatmap, the SNVs/DVGs clustered into five hierarchical groups namely clusters I to V, among which cluster I negatively correlated with cluster II to V, cluster III contained only one SNV (SNV_C1930T), cluster IV showed positive correlations to either cluster II or V, but no considerable correlation was found between cluster II and V. Additionally, the SNVs/DVGs with *p* < 0.0001 in Mann–Whitney *U* test belonged to cluster II, IV, or V, which emphasized their associations with dengue disease severity. As we analyzed the location of SNVs/DVGs within clusters (**Supplementary Table 4**) by Chi-square test, we observed that their clustering did not display considerably association with the genes where SNVs/DVGs located (*p* = 0.324, Chi-square test), suggesting SNVs/DVGs which located in different viral genes might cooperatively contribute to viral fitness and pathogenesis in dengue patients. On the whole, we observed substantially diverse profiles in SNV frequencies and the detected times of DVGs between the mild and severe groups, which might provide valuable intrahost viral population information which associated with disease outcomes.

### ML Model Development and Explainability for Dengue Disease Outcome Classification

**Figure 6:**
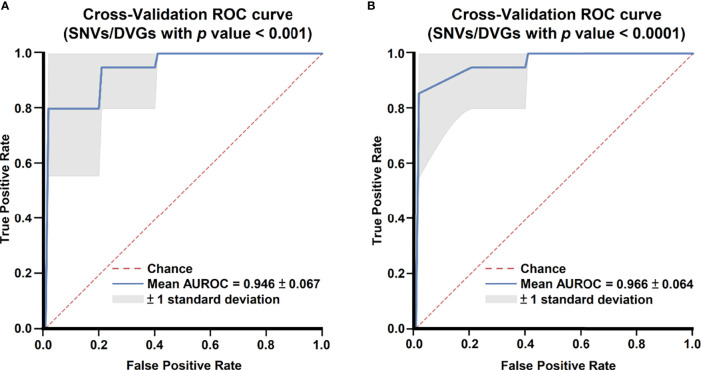
Comparing receiver operating characteristic curves of ML models with 10-fold cross-validation. Mean receiver operating characteristic curves of ML models with selected SNVs/DVGs with **(A)** *p* < 0.001 and **(B)** *p* < 0.0001 in Mann–Whitney U test are shown.

After identifying SNVs and DVGs that are associated with dengue severity, we applied an ML algorithm XGBoost to examine the use of SNVs and DVGs to discriminate diverse disease severity in DENV-2 patients. As feature selection, we chose 44 SNVs with 5 DVGs (*p* < 0.001) and 9 SNVs with 1 DVG (*p* < 0.0001) (**Tables 1**, **2**) to develop ML models, respectively. The performance of developed models was compared using balance accuracy, average precision (AP), and AUROC. Balance accuracy indicated the average recall obtained in each group. AP is a measure that combines recall and precision for ranked retrieval results, while AUROC is a performance measurement for the classification at various threshold settings. Results showed that the model which was developed under feature selection criterion *p* < 0.0001 showed better performance in severe dengue classification (**Table 3** and **Figure 6**). According to ten-fold cross-validation results, we found a good performance for the model in *p* < 0.0001 criterion with 0.970 ± 0.064 in contrast with the model in *p* < 0.001 criterion exhibiting 0.950 ± 0.067 in terms of balance accuracy value (**Table 3**). The model within *p* < 0.0001 criterion showed similar average precision (0.925 ± 0.160 vs 0.925 ± 0.095) (**Table 3**) and higher AUROC values (0.966 ± 0.064 vs. 0.946 ± 0.067) (**Table 3** and **Figure 6**) than the model within *p* < 0.001 criterion, suggesting the successful establishment of an ML model to accurately discriminate the disease outcomes of dengue patients based on 9 SNVs and 1 DVG.

**Table 3:** Comparison of ML models by 10-fold cross-validation for disease severity prediction in DENV 2 infection.

*p* value in Mann–Whitney u test	Selection criteria for SNVs and DVGs	
	*p* < 0.001(44 SNVs/5 DVGs)	*p* < 0.0001(9 SNVs/1 DVG)
Balance accuracy	0.950 ± 0.067	0.970 ± 0.064
Average precision (AP)	0.925 ± 0.095	0.925 ± 0.160
Area under the receiver operating characteristic curve (AUROC)	0.946 ± 0.067	0.966 ± 0.064

To interpret the classification results of the developed ML model, we split the enrolled cases into a 9:1 ratio (training dataset:testing dataset) and developed an ML prediction model. Next, we applied SHAP library to define the importance of each feature and the interplay of feature pairs for the results using a beeswarm plot and a dependent plot in the SHAP program, respectively. The beeswarm plot summarized how each feature generally contributed to classification results, and the dependent plot displayed how feature pairs cooperatively change classification results. SHAP library determines the SHAP values for each feature in all examined datasets. A positive SHAP value (SHAP value > 0) in our classification model indicates that the value of the feature contributes to the increase of probability to severe dengue, and a negative SHAP value (SHAP value < 0) indicates a reduction in the probability.

**Figure 7:**
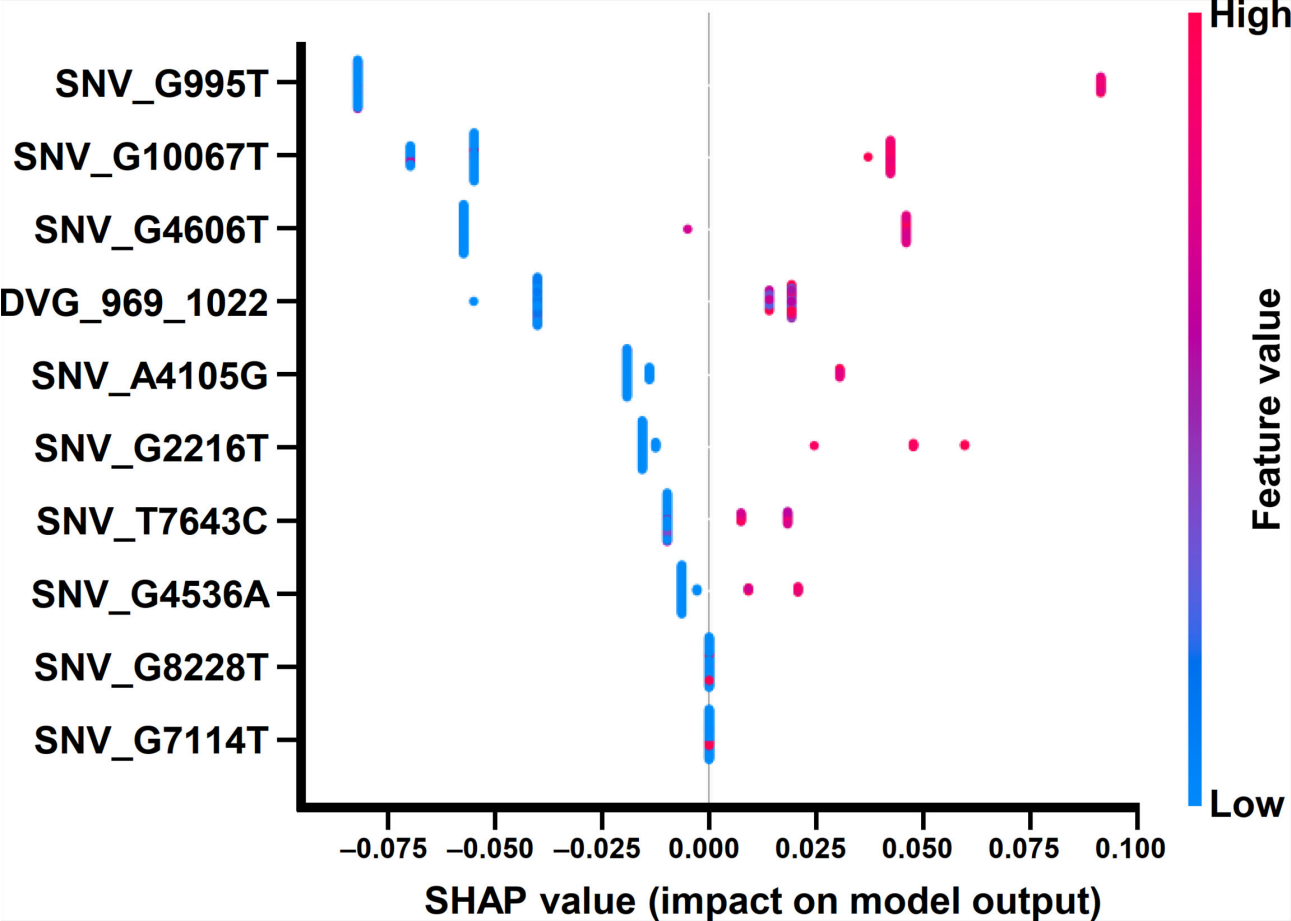
Beeswarm plot of the ML model using SHAP explainer. Beeswarm plot mapping the effects of the indicated features on prognosis outcomes, which assigns a continuous color to the features based on the detection frequency and the number of times each SNV/DVG was selected.

**Figure 8:**
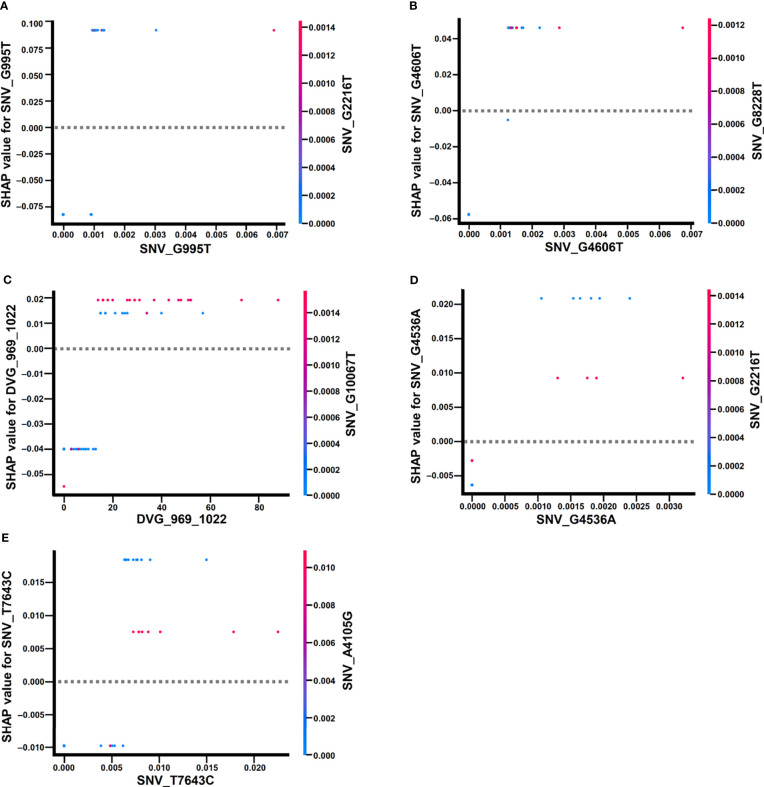
Dependence plots of the ML model using SHAP explainer. Dependence plots of the ML model showed the potential cooperative effects of feature pairs on prognosis outcomes. For each feature, its most associated feature was chosen to be analyzed using dependence plots. The feature pairs were **(A)** SNV_G995T and SNV_G2216T, **(B)** SNV_G4606T and SNV_G8228T, **(C)** DVG_969_1022 and SNV_G10067T, **(D)** SNV_G4536A and SNV_G2216T, and **(E)** SNV_T7643C and SNV_A4105G.

In the beeswarm plot of ML model, features were ordered from the top to the bottom of plot according to their importance. The beeswarm plot of ML model showed that the top eight important features were the frequency of SNP_G995T, SNV_G2216T, SNV_A4105G, SNV_G4536A, SNV_G4606T, SNV_T7643C, SNV_G10067T, and the detection times of DVG_969_1022 (**Figure 7**), all of which showed a SHAP value shift from negative to positive as their values increased. In contrast with the top eight features, another two features, the frequency of SNP_G7114T and SNP_G8228T, did not show a SHAP value shift as their values changed. We next examined the interaction effects between the top eight important features in the model using dependent plots (**Figures 8A–E**). The dependent plots showed how the model depended on a given feature and for each feature, picked another feature with which it had the strongest interaction. Similar to the beeswarm plot, the SHAP values increased from negative to positive in all the dependent plots of the feature pairs, and various interaction effects on SHAP values were observed among the feature pairs. In the dependent plots of SNV_G995T/SNV_G2216T and SNV_G4606T/SNV_G8228T pairs, the SHAP values changed without any considerable effect exerted by the feature pair cooperation (**Figures 8A, B**). In contrast, we observed that DVG_969_1022/SNV_G10067T exhibited a synergistic effect on SHAP values, whereas positive SHAP values elevated as both the detection time of DVG_969_1022 and the frequency of SNV_G10067T increased (**Figure 8C**). The SNV_G4536A/SNV_G2216T pair and SNV_T7643C/SNV_A4105G pair displayed antagonistic effects. The high frequency of SNV_G2216T and SNV_A4105G reduced the positive SHAP values which were resulted by SNV_G4536A and SNV_T7643C, respectively (**Figures 8D, E**). Taken together, positive SHAP values were observed when the frequency of both SNVs in a pair increased; however, the SNVs/DVG pairs could still synergistically or antagonistically affect the positive SHAP values, suggesting their various cooperative effects on the result of the ML model classification. We determined that the expression abundance of 9 SNVs and 1 DVG in the sera was associated with disease severity of DENV-2 patients. Combining with the ML algorithm, we developed an accurate classification model for dengue severity and identified the various interactive effects of SNVs/DVG on dengue severity.

## Discussion

Several reports have demonstrated potential arbovirus hospitalization based on various risk factors by utilizing ML algorithms (Sippy et al., 2020; Ozer et al., 2021). Our previous study developed a severe dengue prognosis model for rapid triage using demographic information and dengue antigen/antibody rapid test results from dengue patients (Huang S. W. et al., 2020); however, 75% (18 in 24 false cases) of anti-dengue antibody negative cases were incorrectly discriminated. Since the sera samples were collected immediately upon patient arrival in our hospital and DENV viremia occurs for 3–5 days prior to fever onset and continues for approximately 5 days into febrile illness, the majority of false discriminated patients were experiencing the early acute phase of dengue disease in primary infection (Hunsperger et al., 2016). Here, we provided a potential improvement by applying intrahost viral population in the sera of primary dengue infection patients to predict severe dengue with ML. Similar to the NS1 antigen, viral RNA genome is another abundant marker in patient serum after the day of disease onset, which was widely applied in the dengue patient’s diagnosis upon their hospital arrival. By analyzing the abundance of the SNVs and DVGs in the sera, we found hundreds of intrahost viral variants significantly associated with severe dengue. Using the ML algorithm, we developed an accurate model which proved the potency that SNVs/DVGs could distinguish mild and severe cases according to the frequency of the 9 selected SNVs and the detection times of 1 DVG. Model interpretation showed that an increase in the selected SNV frequencies and the selected DVG detection times positively correlated with severe disease. Although the roles of identified SNVs and DVG in the dengue pathogenesis require further investigation, our study provided a new insight into the association of intrahost viral population with severe dengue and accessed the potency of SNVs and DVGs to be utilized for dengue severity prognosis by applying the compositions of mutant spectrum in intrahost viral population and the ML methodology.

Our intrahost population results displayed a series of SNVs and DVGs that associated with disease outcomes among DENV-2 patients (**Tables 1**, **2**). We suggested that the SNVs and DVGs can be transmissible and adapted in human-mosquito transmission cycles, which might exhibit higher fitness, pathogenesis, or virulence in the human patients. Most viral SNVs and DVGs detected from the sera of dengue patients in our study were suggested to be packaged as virions due to the abundant RNase existing in sera, which can degrade naked RNA (Blank and Dekker, 1981). Thus, the virion-packaged SNVs and DVGs in the blood of dengue patients might be transmissible among human hosts by the *Aedes* mosquitos biting, although few naked viral genomes were possibly released into the blood stream from lysed DENV-infected tissues/cells. Furthermore, mutant variants including SNVs and DVGs have been reported to long-term transmit in human-mosquito transmission cycles (Aaskov et al., 2006) and retain in variant reservoir. Recently, several SNVs have been identified to generally appear among Brazilian DENV-2 patients, and their frequency correlated with disease severity (Torres et al., 2021). When we further analyzed all DENV-2 sequences published in the GenBank database (**Supplementary Table 5**), we observed that the nucleotide polymorphisms appeared at the position of the SNVs which associated with dengue outcomes in our study, including SNV_G995T, SNV_G2216T, SNV_A4105G, SNV_G4606T, SNV_T7643C, SNV_G8228T, and SNV_G10067T, among which SNV_G995T, SNV_G2216T, SNV_A4105G, and SNV_G8228T had nucleotide changes identical to that identified in our recent study. Additionally, SNV_G2216T was further defined in two natural DENV-2 sequences from mosquitos, suggesting that our identified SNVs might be neutral for DENV evolution, which potentially retained in viral reservoir in human-mosquito transmission cycles (**Supplementary Table 5**). In natural human infection, the SNVs/DVGs in viral repertoire might benefit virus to adapt in susceptible tissues/cells in intrahost evolution and individually or cooperatively elevate viral virulence and/or immunopathogenesis in dengue patients. Nonetheless, we did not rule out the possibility that certain SNVs/DVGs were essential for DENV-2 to colonize in susceptible tissues/cells, which adapted separately among patients with dengue disease without transmission in human-mosquito cycles. Since only 2 to 3 µL of blood is withdrawn during a female mosquito bite, the variants with low frequency are prone to be extinct from the viral repertoire in human-mosquito transmission (Ogunrinade, 1980; Lequime et al., 2016). Further studies are necessary to investigate whether the SNVs/DVGs are transmitted between mosquitoes and humans and whether they change viral virulence and pathogenesis *in vitro* and *in vivo*.

Since the significant high proportion of SNVs identified using the Mann–Whitney *U* test contributed to amino acid changes between mild and severe dengue patient groups, amino acid residues at the positions of these SNVs were suggested to shaped under certain selection pressures, such as success replication in the specific tissues of human hosts or immune selection by human B cell and T cells due to the abundant SNVs located in the immunodominant dengue protein E and NS3 (Simmons et al., 2005; Dejnirattisai et al., 2010; Rivino et al., 2013; de Alwis et al., 2014), which might lead to viral protein function alternation. Upon explaining our ML model, seven out of the top eight important features to discriminate dengue disease outcomes are SNVs with amino acid residue substitutions, which located at the coding regions of structural E protein and non-structural NS2A, NS3, and NS5 proteins.

The E protein is important to the initiation of DENV infection through its functions in host receptor attachment, virus internalization, and viral RNA release. As triggering by low pH in viral entry, the E protein makes a conformation change from E-dimer to E-trimer and then release viral genome into cells. The N-terminus of E protein (residue 1-394) is a soluble ectodomain with domains I, II, and III, whereas the C-terminus of E protein is the stem (residue 395-448) and transmembrane domains (residue 449-495). The SNV_G995T, which identified in our study, resulted in the substitution at the 20^th^ residue of E protein (E-20 residue) and located at the B_0_ β-sheet of domain I in E protein ectodomain. Crystal and Cryo-EM structures of E protein showed that the B_0_ β-sheet of domain I did not expose to outer surface because this structure neighbors to the membrane side of the DENV virions as an E-dimer and located at the trimer contact region as an E-trimer (Modis et al., 2004; Zhang et al., 2013). The E-427 residue of which codon at SNV_G2216T located at the stem domain. Among flaviviruses sequences, the stem domain is highly conserved (Klein et al., 2013) and were further applied as broad-spectrum antiviral peptides against DENV (Chew et al., 2017). Although more evidence is required, we suggest that the E-20 and E-427 residues are more likely associated with immunodominant T cell epitope due to their inaccessibility for neutralization antibody of DENV.

In addition to the 2 SNVs locating at E protein coding region, 5 SNVs contributed to amino acid substitutions at nonstructural NS2A, NS3, and NS5 proteins. NS2A is associated with the endoplasmic reticulum membrane and plays role in virus replication, virion assembly, and immune evasion (Gopala Reddy et al., 2018). The SNV_A4105G caused amino acid substitution at NS2A-210 residue, which is located at the non-transmembrane C-terminal end (residue 210-218) close to the cleavage junction between NS2A and NS2B; however, the role C-terminal end peptide, including NS2A-210 residue, in DENV replication warrants further investigations. NS3 plays an essential role in virus polyprotein cleavage and viral RNA replication. NS3 protein includes the N-terminus domain (residue 1-168) and the C-terminus domain (residue 180-618) which are linked by a linker peptide (residue 169-179). Both of the SNV_G4536A and SNV_G4606T located at the coding region of N-terminal NS3 protein, which has protease activity with NS2B as a cofactor. The NS3-5 residue of which codon at SNV_G4536A is close to the cleavage site of NS2B which has been applied for the design of DENV protease inhibitors (Majerova et al., 2019). The NS3-29 residue of which codon at SNV_G4606T located at a conserved hydrophobic loop (residue 29 to 32). Structural model analysis indicated the potency of the loop attribute to the contact of NS2B-NS3 complex and membrane (Luo et al., 2010). The two residues at NS3 protein, as part of the structure in NS2B/NS3 complex, implied their potential roles in DENV NS3 protease cleavage.

NS5 protein possesses multiple enzymatic activities involved in viral RNA capping and replication. It is composed of the N-terminal methyltransferase (MTase) domain and the C-terminal RNA-dependent RNA polymerase (RdRp) domain that are connected by the linker region. The NS5-25 residue of which codon at SNV_T7643C located at the GTP-binding site of the MTase domain. The NS5-F25A substitution resulted in the reduction of RNA and GTP binding affinities, which are important in viral RNA capping (Henderson et al., 2011). In addition, NS5-25 is at the NS5 and human STAT2 binding sites (Wang et al., 2020). The binding of NS5 to STAT2 suppresses the downstream interferon responses that help the viral infection. The NS5-833 residue of which codon at SNV_G10067T located at the thumb subdomain of the RdRp domain. The NS5-833 residue has been identified at the binding pocket of several RdRp inhibitors, implied its importance in DENV RdRp activity. Together, protein residues at these seven SNVs may have potential roles in regulating viral protein interaction, virus infection, and host immune escape although they have yet been identified as determinants in viral pathogenesis and fitness. Future studies using mutagenesis with reverse genetics of DENV may be needed to determine whether the SNVs solely or synergistically affect viral protein functions and virus properties. Since the E-427 (Chew et al., 2017), NS3-5 (Majerova et al., 2019), and NS5-833 (Nascimento et al., 2021) residues have been applied as target regions when anti-DENV agents designs, it might be necessary to verify the potency of anti-DENV agents as treatments when developing novel drugs in severe dengue patients in the future.

The DVG_969_1022 was the only DVG selected from the top eight important features to discriminate dengue disease outcomes in our ML model. DVG has been well-documented in various RNA virus infections to induce strong interferon response, which activates RIG-I-like receptors and increases the expression of IFN and proinflammatory cytokines such as IL-1α, TNF, and IL-6 (Yount et al., 2006; Tapia et al., 2013; Sun et al., 2015; Ho et al., 2016). By interfering with standard virus replication, which delays the spread of virus and aids the host to develop further immune response (Dimmock and Easton, 2014). Recently, Rezelj et al. (2021) showed that the prM-E-NS1 region truncated genome inhibited Zika virus replication in various cells in an RNAi dependent manner, which can be reduce viral virulence in mice and dissemination/transmission in mosquitoes (Rezelj et al., 2021). Nonetheless, our recent study found that the associated with abundance of DVG_969_1022, which had truncated E protein region of viral genome, significantly increased virulence among primary DENV-2 infected patients. We suggested that the virulence impacts of DVGs on dengue disease outcome in our study resulted from the immunopathogenesis of the DVG_969_1022 because the truncation of DVG_969_1022 causes the remaining open reading frame to be out of frame. DVGs were strong stimulators for inducing abundant proinflammatory cytokines including IL-6 (Butthep et al., 2012; Liao et al., 2015). The overproduced IL-6 might enhance production of anti-platelet or anti-endothelial cell autoantibodies, elevated levels of tPA, and deficiency in coagulation (Lei et al., 2001), which may explain higher occurrence levels of DVG_969_1022 in severe dengue patients.

Although our developed ML model can accurately classify mild and severe dengue cases in our dataset, several issues will need be explored before applying our ML model for dengue disease prognosis. First, the predominant strains of DENV outbreaks or epidemics varied in different regions. In this study, we retrospectively included 65 patients from the 2015 Taiwan outbreak, but all patients were infected by the Cosmopolitan genotype strains of DENV-2 in a close cluster. In addition to potential bias arising due to a single predominant strain in the outbreak, elderly individuals constituted the major age group among the patients with severe dengue in the Taiwan outbreak, in contrast to the severe cases in children in southeastern Asia (Hoang et al., 2010) and South America (Allonso et al., 2014) as previously described (Tsai et al., 2016; Huang S. W. et al., 2020). Our dataset only came from the patients who seek medical advice in the hospital, which might exclude the mild cases who did not go to the hospital and resulted in the potential loss of true intrahost dynamics among mild cases. Further investigation is needed to determine whether the prognosis model can be applied in other patients with dengue, infected by other genotypes/serotypes, having younger ages, or the mild cases who did not seek medical advice in the hospital. Second, our findings require validation in a larger prospective cohort before the prognostic model could be applied in a clinical setting, as our study is a retrospective study with a limited number of enrolled patients. Third, our ML model, according to variants detection by deep sequencing, exhibited high performance to triage the mild and severe dengue patients; however, the long consuming time and high costs of whole processes using deep sequencing might limit its clinical applications in real world. Alternative detection methods for SNVs/DVGs such as digital RT-PCR platforms (Uchiyama et al., 2016) might need to be developed to rapidly detect the severity-associated variants with low appearing frequency in the sera of dengue patients with lower costs for clinical application; however, the detection sensitivity of these platforms still needs verification.

In conclusion, we defined the SNVs and DVGs of which the abundance in sera from DENV-2 infected patients in primary infection highly associated with severe dengue disease. The identified SNVs/DVG could accurately predict the disease outcomes of DENV-2 infected patients by using an ML model. We believe that our prognostic models would help clinicians to efficiently triage patients with DENV-2 infection upon their hospital arrival after a rapid assay for SNVs/DVGs detection is developed.

## Data Availability Statement

The deep sequencing reads of DENV-2 were deposited in the NCBI Sequence Read Archive (accession number SRR16924914 and SRR16943989 to SRR16944052) under the BioProject ID PRJNA779757.

## Ethics Statement

The studies involving human participants were reviewed and approved by Institutional Review Board of National Cheng Kung University Hospital. Written informed consent for participation was not required for this study in accordance with the national legislation and the institutional requirements.

## Author Contributions

S-WH, S-JH, and Y-FW contributed to conception and design of the study. S-WH, S-JH, Y-FW, H-PT, W-CK, and J-RW organized the database. S-WH and S-JH performed the statistical analysis. S-WH and S-JH wrote the first draft of the manuscript and Y-FW, H-PT, W-CK, and J-RW review and edit of the manuscript. All authors provide resources and contributed to manuscript revision, read, and approved the submitted version.

## Funding

J-RW received funding from the Ministry of Science and Technology (grant nos. MOST-110-2327-B-006-005, 10A1-MRGP05-035; https://www.most.gov.tw/). SWH received funding from the National Health Research Institutes (grant nos. MR-107-PP-17, MR-108-GP-04, MR-109-GP-04, and MR-110-GP-04; http://www.nhri.org.tw/). The funders had no role in the study design, data collection, and analysis, decision to publish, or preparation of the manuscript.

## Conflict of Interest

The authors declare that the research was conducted in the absence of any commercial or financial relationships that could be construed as a potential conflict of interest.

## Publisher’s Note

All claims expressed in this article are solely those of the authors and do not necessarily represent those of their affiliated organizations, or those of the publisher, the editors and the reviewers. Any product that may be evaluated in this article, or claim that may be made by its manufacturer, is not guaranteed or endorsed by the publisher.
