# Supplementary material for: Assessment of the Risk of Severe Dengue Using Intrahost Viral Population in Dengue Virus Serotype 2 Patients via Machine Learning
Source: Front Cell Infect Microbiol. 2022 Feb 10;12:831281. doi: 10.3389/fcimb.2022.831281 (PMC8866709; doi:10.3389/fcimb.2022.831281)
Supplement: Supplementary file 6 [file Presentation_1.pptx]

## Slide 1
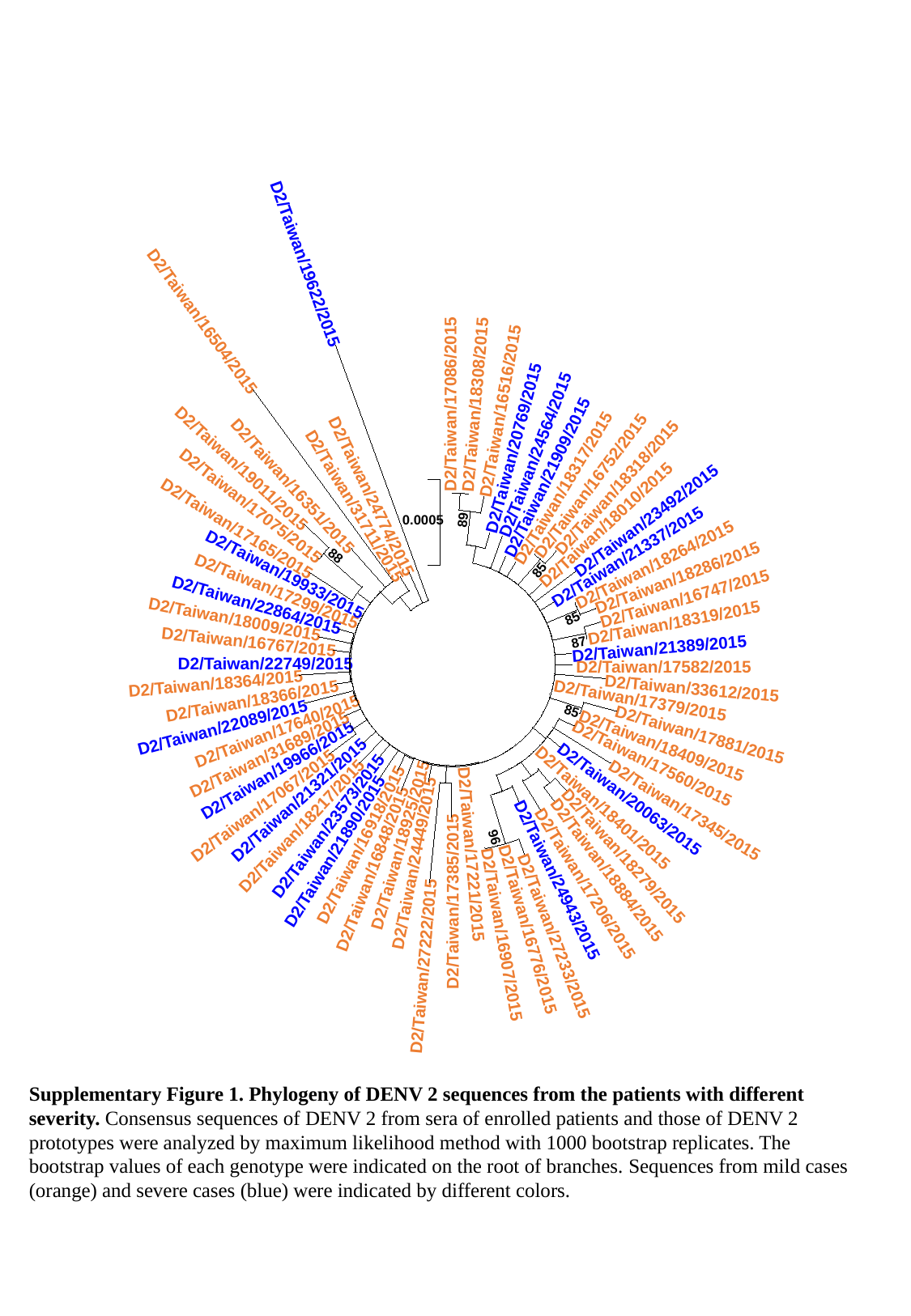

D2/Taiwan/19622/2015
D2/Taiwan/16504/2015
D2/Taiwan/17086/2015
D2/Taiwan/18308/2015
D2/Taiwan/16516/2015
D2/Taiwan/20769/2015
D2/Taiwan/24564/2015
D2/Taiwan/21909/2015
D2/Taiwan/16752/2015
D2/Taiwan/18318/2015
D2/Taiwan/18317/2015
D2/Taiwan/23492/2015
89
D2/Taiwan/18010/2015
D2/Taiwan/21337/2015
D2/Taiwan/18264/2015
85
D2/Taiwan/18286/2015
D2/Taiwan/16747/2015
85
D2/Taiwan/18319/2015
87
D2/Taiwan/21389/2015
D2/Taiwan/17582/2015
D2/Taiwan/33612/2015
D2/Taiwan/17379/2015
85
D2/Taiwan/17881/2015
D2/Taiwan/18409/2015
D2/Taiwan/17560/2015
D2/Taiwan/20063/2015
D2/Taiwan/18401/2015
D2/Taiwan/17345/2015
96
D2/Taiwan/17221/2015
D2/Taiwan/18279/2015
D2/Taiwan/18884/2015
D2/Taiwan/24943/2015
D2/Taiwan/17206/2015
D2/Taiwan/16776/2015
D2/Taiwan/16907/2015
D2/Taiwan/27233/2015
D2/Taiwan/19011/2015
D2/Taiwan/16351/2015
D2/Taiwan/24774/2015
D2/Taiwan/17075/2015
D2/Taiwan/31711/2015
0.0005
D2/Taiwan/17165/2015
88
D2/Taiwan/19933/2015
D2/Taiwan/17299/2015
D2/Taiwan/22864/2015
D2/Taiwan/18009/2015
D2/Taiwan/16767/2015
D2/Taiwan/22749/2015
D2/Taiwan/18364/2015
D2/Taiwan/18366/2015
D2/Taiwan/22089/2015
D2/Taiwan/17640/2015
D2/Taiwan/31689/2015
D2/Taiwan/19966/2015
D2/Taiwan/21321/2015
D2/Taiwan/17067/2015
D2/Taiwan/18217/2015
D2/Taiwan/23573/2015
D2/Taiwan/16918/2015
D2/Taiwan/18925/2015
D2/Taiwan/21890/2015
D2/Taiwan/24449/2015
D2/Taiwan/16848/2015
D2/Taiwan/17385/2015
D2/Taiwan/27222/2015
Supplementary Figure 1. Phylogeny of DENV 2 sequences from the patients with different severity. Consensus sequences of DENV 2 from sera of enrolled patients and those of DENV 2 prototypes were analyzed by maximum likelihood method with 1000 bootstrap replicates. The bootstrap values of each genotype were indicated on the root of branches. Sequences from mild cases (orange) and severe cases (blue) were indicated by different colors.

## Slide 2
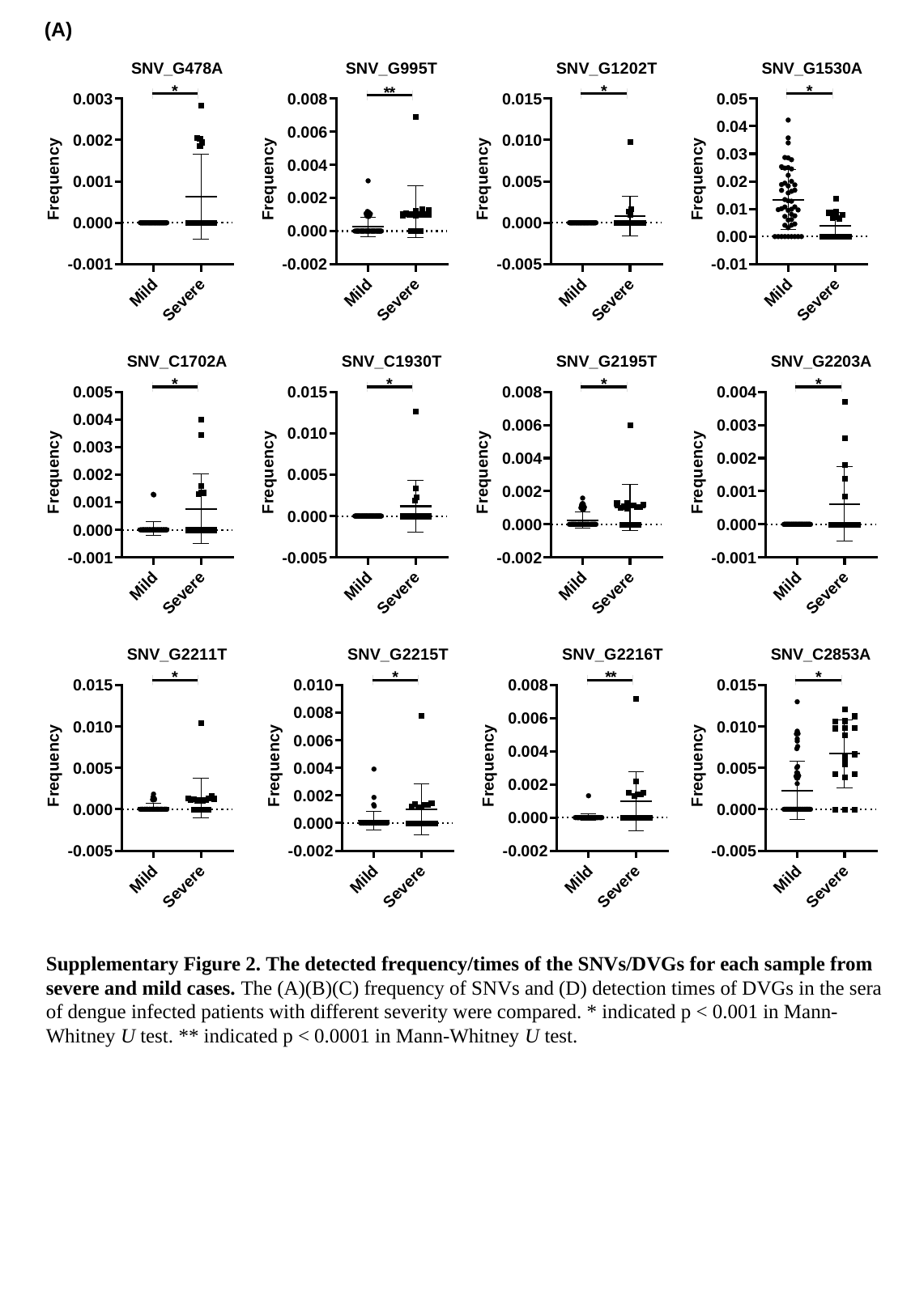

(A)
Supplementary Figure 2. The detected frequency/times of the SNVs/DVGs for each sample from severe and mild cases. The (A)(B)(C) frequency of SNVs and (D) detection times of DVGs in the sera of dengue infected patients with different severity were compared. * indicated p < 0.001 in Mann-Whitney U test. ** indicated p < 0.0001 in Mann-Whitney U test.

## Slide 3
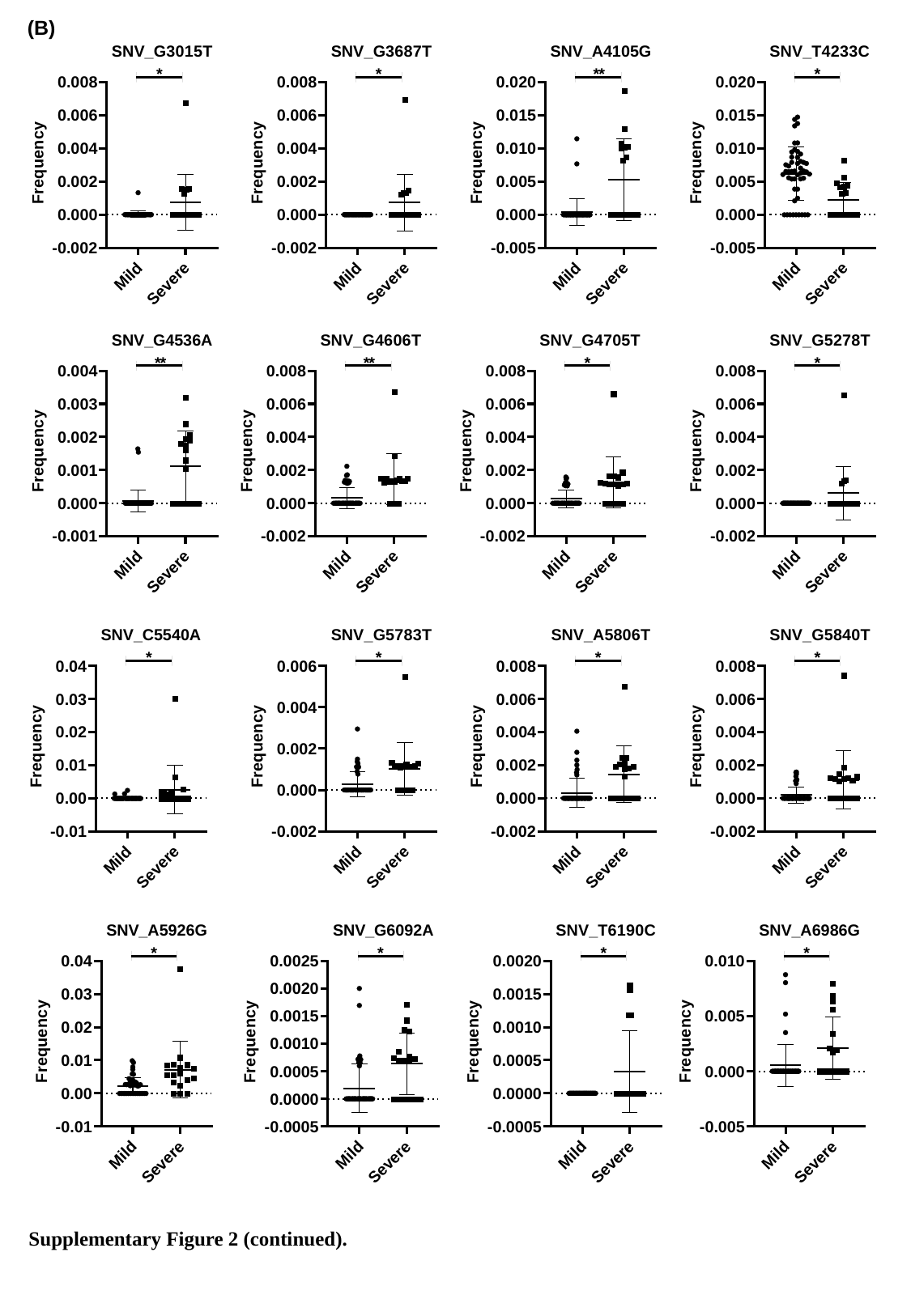

(B)
Supplementary Figure 2 (continued).

## Slide 4
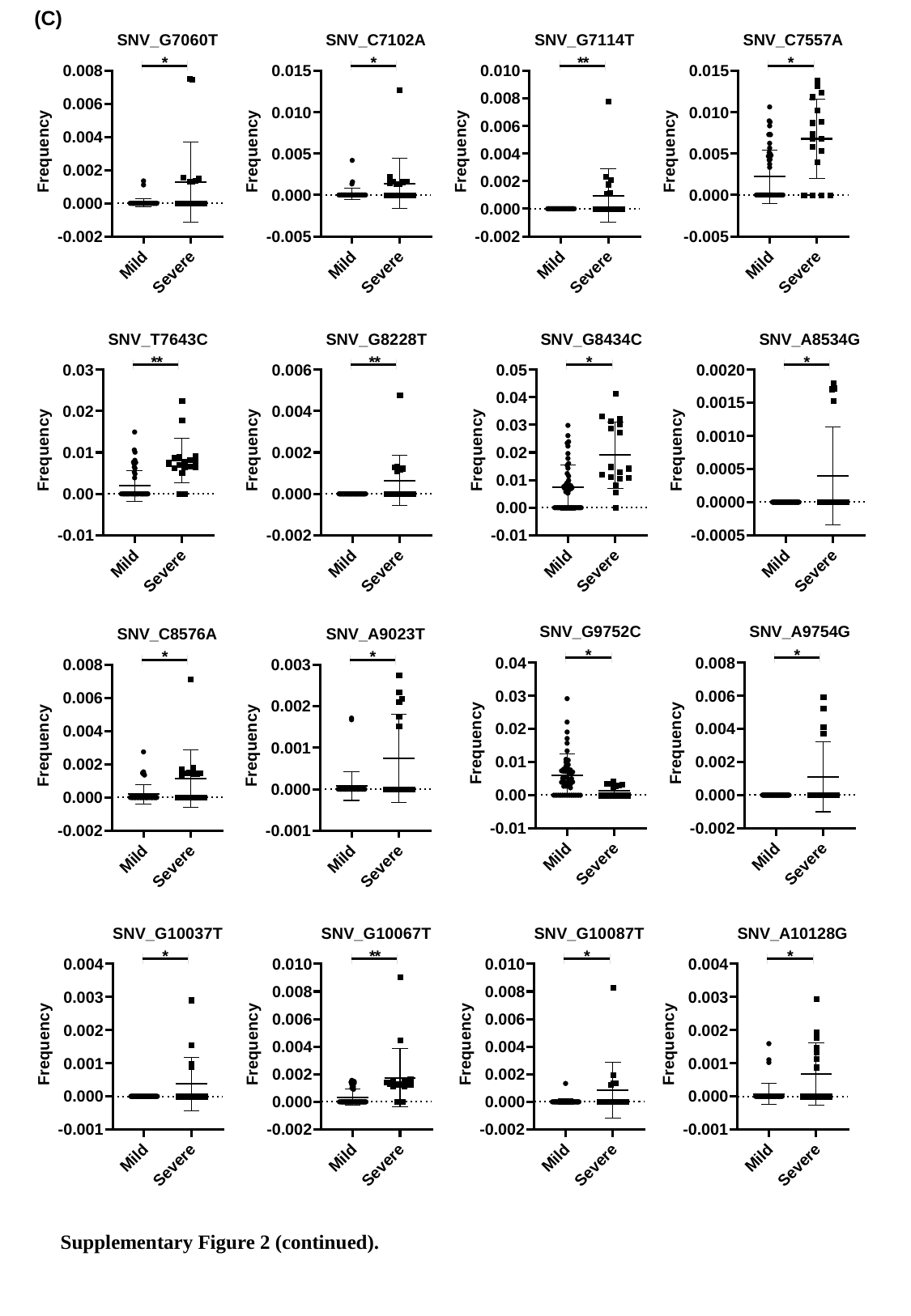

(C)
Supplementary Figure 2 (continued).

## Slide 5
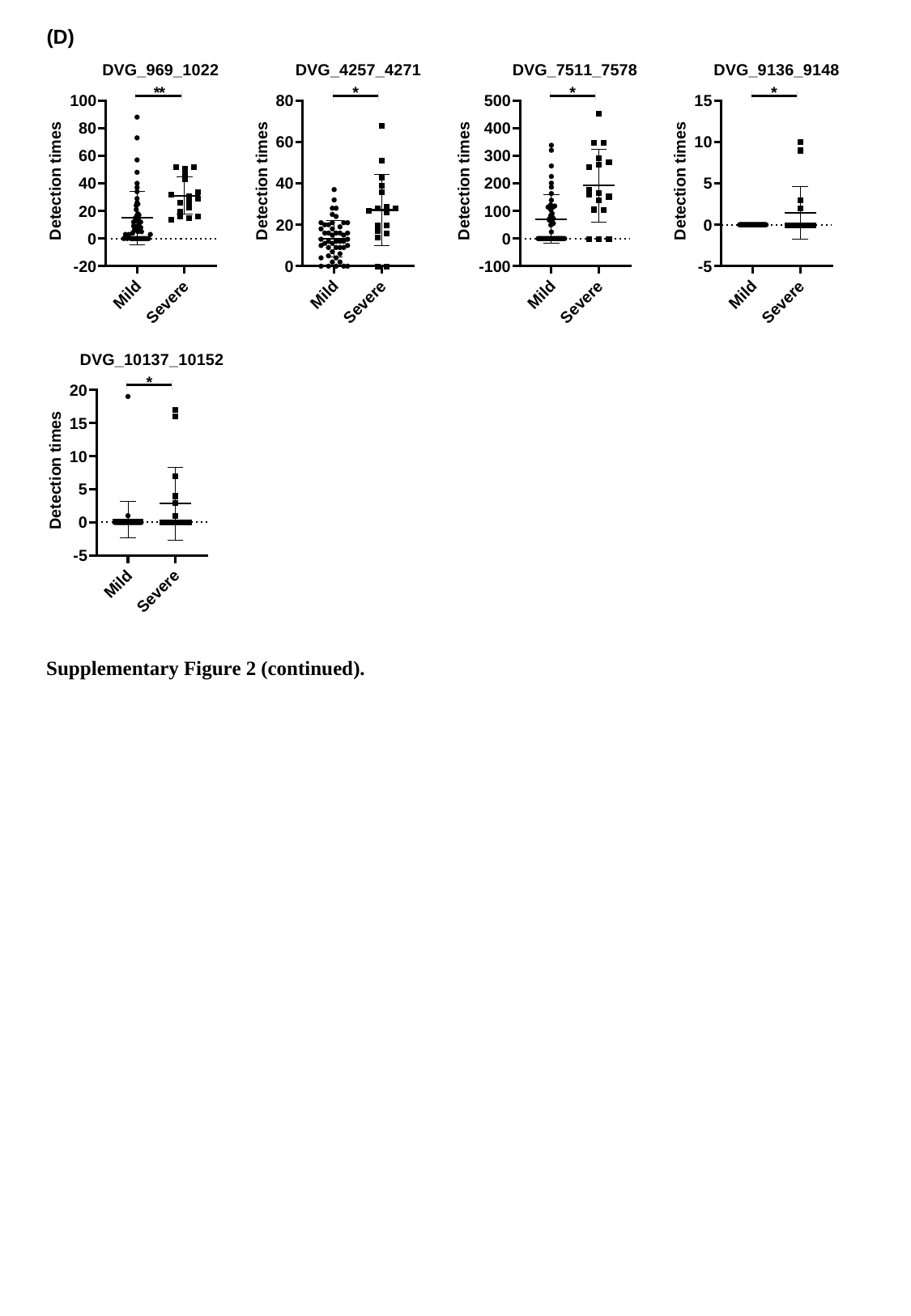

(D)
Supplementary Figure 2 (continued).
